# UPLC-QTOF/MS-Based Lipidomic Profiling of Liver Qi-Stagnation and Spleen-Deficiency Syndrome in Patients with Hyperlipidemia

**DOI:** 10.1155/2018/4530849

**Published:** 2018-08-30

**Authors:** Piao Shenghua, Tan Shuyu, Li Kunping, Zhan Huixia, Xiao Xue, Guo Jiao

**Affiliations:** ^1^Key Unit of Modulating Liver to Treat Hyperlipemia SATCM (State Administration of Traditional Chinese Medicine), Guangdong Pharmaceutical University, Guangzhou Higher Education Mega Centre, Guangzhou 510006, China; ^2^Guangdong TCM Key Laboratory against Metabolic Diseases, Guangdong Pharmaceutical University, Guangzhou Higher Education Mega Centre, Guangzhou 510006, China; ^3^Guangdong Province Research Centre for Chinese Integrative Medicine against Metabolic Disease, Guangdong Pharmaceutical University, Guangzhou Higher Education Mega Centre, Guangzhou 510006, China

## Abstract

Hyperlipidemia is a common disease caused by abnormal plasma lipid metabolism. Lipidomics is a powerful and efficient technology to study the integration of disease and syndrome of Chinese medicine. This study investigated specific changes in lipid metabolites from hyperlipidemia patients with syndrome of liver qi-stagnation and spleen-deficiency (SLQSD). Lipid profiles in plasma samples from 29 hyperlipidemia patients including 10 SLQSD and 19 non-SLQSD and 26 healthy volunteers (NC) were tested by UPLC-QTOF/MS. PLS-DA analysis and database searching were performed to discover differentiating metabolites. Differences in lipid metabolites between hyperlipidemia and healthy people mainly include phosphatidylcholines, phosphatidylethanolamines, phosphatidylglycerols, and ceramides. Hyperlipidemia patients with SLQSD and non-SLQSD could be differentiated by using identified lipid metabolites including phosphatidylcholines, phosphatidylethanolamines, phosphatidylinositols, triglycerides, diacylglycerols, lysophosphatidylethanolamines, sphingomyelins, lysophosphatidylcholines, and lactosylceramides. There were significant differences of lipid metabolism between between different syndromes of the same disease such as hyperlipidemia which showed significant differences between SLQSD and non-SLQSD.

## 1. Introduction

Hyperlipidemia is a common disease caused by abnormal plasma lipid metabolism and is considered a high independent risk factor for atherosclerotic cardiovascular and cerebrovascular disease such as coronary heart disease and stroke. In traditional Chinese medicine (TCM), hyperlipidemia is called lipid turbidity and is treated based on syndrome differentiation. With the transformation of life style hyperlipidemia showed a trend in young people and the syndrome of Chinese medicine changed from spleen-kidney deficiency to stagnation of liver qi and spleen deficiency (SLQSD) [1, 2]. The syndrome of liver depression and spleen deficiency is the main syndrome of hyperlipidemia [3, 4].

The investigation of syndrome essence is a key challenge in the field of Chinese medicine. Until now, due to limitations of the methods available, the progress towards understanding such complicated systems has been slow. As the most important section in the TCM system, syndrome differentiation based on the clinical manifestations from traditional four diagnostic methods naturally has biological foundation. Except for total cholesterol (TC), total triglyceride (TG), low density lipoprotein cholesterin (LDL-C), and high-density lipoprotein cholesterol (HDL-C), does hyperlipidemia have any difference in lipid metabolites between syndrome of SLQLD and non-SLQLD?

Metabolomics has been widely applied to disease biomarker discovery, drug mechanism evaluation, and pharmacological activity and toxicity evaluation especially in traditional Chinese medicine in both animal models and clinical studies [5–10]. As a new branch of metabolomics, Lipidomics is an emerging discipline that aims to systematically analyze various lipids used in various diseases including hyperlipidemia to reveal the regulation of endogenic metabolites [11–15]. Ultra performance liquid chromatography coupled with quadrupole time-of-flight mass spectrometry (UPLC-QTOF/MS) is most suitable for lipidomics, especially for untargeted lipid profiles [16–19]. The holistic view and system theory of Chinese medicine coincide with the systematical study of lipid metabolites. UPLC-Q-TOF/MS-based lipidomics has been widely applied to the hyperlipidemia and therapeutic effect of TCM on hyperlipidemia [15, 20–22]. In the current study, lipidomics was used to explore the lipid metabolites of hyperlipidemia patients with syndrome of stagnation of liver qi and spleen deficiency.

## 2. Materials and Methods

### 2.1. Diagnostic Criteria and Syndrome Differentiation

The diagnostic criteria for hyperlipidemia were mainly obtained from “Guideline of Chinese adult dyslipidemia Prevention and Treatment (2007) [23].” Syndrome differentiation criteria were mainly obtained from the textbook “Diagnostics of Traditional Chinese Medicine [24]” and “TCM clinical diagnostic and treatment practices (2002) [25].” Criteria of syndrome of stagnation of liver qi and spleen deficiency included main symptoms, secondary symptoms, and syndrome determination. The characteristics of main symptoms were emotional depression or irritability, flank swelling and pain, poor appetite, loose stools, string, or thin pulse. The characteristics of secondary symptoms were paleness, tiredness and not wanting to talk, frequent sighing, abdominal painful distension, obesity, uncomfortable loose bowels or alleviation of abdominal pain after defecation, pale tongue, and white tongue coating. The characteristics of syndrome determination were three or more mains syndromes or two main syndromes and three or more secondary symptoms and with the reference of tongue and pulse.

### 2.2. Patient Selection

All subjects were recruited from the First Affiliated Hospital of Guangdong Pharmaceutical University. There were 29 patients with primary hyperlipidemia including 10 cases with the symptom of SLQSD and 19 cases with non-SLQSD. Control group (normal group) consisted of 26 healthy volunteers with no cold and other acute diseases. Individuals who volunteered to be a subject signed an informed consent form; those who were aged 30 to 70 years and met the diagnostic criteria for hyperlipidemia were included. Secondary hyperlipidemic patients were excluded. Patients with colds, acute gastroenteritis, and other acute diseases in the survey period which interfered with the judgment of candidates were not included. Patients with cerebral infarction, myocardial infarction, other serious diseases, and mental illness or who could not cooperate with the investigation were excluded.

### 2.3. Reagents

HPLC grade acetonitrile was purchased from Merck Company. HPLC grade formic acid was purchased from Dima Company. HPLC grade ammonium acetate and chloroform was purchased from Tianjin Damao Company. HPLC grade methanol was purchased from B&J Company.

### 2.4. Sample Collection and Preparation

Plasma samples were taken after having fasted for more than 12 hours. The next morning 2 mL blood samples was collected from their median cubital vein and stored in 4 mL EDTA microcentrifuge tubes. The samples were centrifuged at 3,000×g for 10 min at 4°C. Plasma was separated and kept frozen at –80°C until analysis.

A 0.1 mL aliquot of each plasma sample was transferred to 1.5 mL polypropylene tubes with a fixed amount of 0.3 mL 2:1 (v/v) CHCl_3_:MeOH. The mixture was settled at room temperature for 5s, and then 75 *μ*L ultra high purity water had been added to the tube and vortex-mixed. The mixture was centrifuged at 10,000 rpm for 5 min at 4°C. The under layer was transferred to another polypropylene tube and evaporated to dryness at room temperature under nitrogen gas. The final residue was redissolved in 120 *μ*L acetonitrile and then was centrifuged at 12000 rpm for 10 min. The supernatant was subjected to UPLC-QTOF/MS analysis.

### 2.5. UPLC Conditions

The samples were analyzed by a Waters Acquity Ultra Performance LC system (Waters, USA) equipped with Waters Xevo™ G2 QTof MS. Chromatographic separation was carried out at 30°C on an Acquity UPLC™ BEH C_18_ (10 × 50 mm). Injection volume was 5 *μ*L. The total flow rate was 0.4 mL/min. The sample chamber temperature was kept at 4°C. The mobile phase consisted of acetonitrile water (0.1% formic acid, 1 mol/L ammonium acetate). The linear solvent gradient was shown in Table 1.

### 2.6. Mass Spectrometry

The mass spectrometric detection was conducted by Q-TOF MS system. ESI ion source was used in both positive and negative ion modes and centroid mode was used to get signal acquisition and did the real-time simultaneous Lock-Mass mass correction with the correction fluid being chloramphenicol (500 pg/*μ*L). Its precise charge to mass ratio was [M + H]^+^ = 345.0021 and [M-H]^−^ = 321.0045, respectively, in both positive and negative ion modes. Mass range was 300-1200m/z. In positive and negative ion modes MS conditions were as follows: capillary cone: 3200 V; sample cone: 39 V; extraction cone: 2.0 V; source temperature: 120°C; desolvation temperature: 250°C; cone gas: 60 L / hr; desolvation gas: 800 L / hr; ion energy: 1.0 V; collision energy: 10 V.

### 2.7. Statistical Analysis

The raw data were processed using the Micromass MarkerLynx Applications Manager version 4.0 (Waters Corp., Milford, USA). This application manager incorporates a peak deconvolution package that allows detection of the mass, retention time, and intensity of the peaks eluting in each chromatogram. The area of each peak, after being recognized and aligned, was normalized to the summed total ion intensity of each chromatogram. The resulting three-dimensional data, peak number (RT-m/z pair), sample name, and normalized ion intensity were introduced to SIMCA-P 10.0 software package (Umetrics, Umea, Sweden) for PCA and PLS-DA. Mean centered was used for data scaling and centering. ANOVA was performed in succession to reveal the statistical differences for the variables normal group, hyperlipidemia with syndrome of SLQSD group, and hyperlipidemia with syndrome of non-SLQSD group. The homogeneity of the variance was tested before ANOVA analysis. For identification of potential markers, the following database has been used: http://www.lipidmap.jp. The significance of variation between groups in data of biological parameters was determined using SPSS for non-parametric tests by Excel 2003 (Microsoft, USA). According to the variable importance in the projection (VIP) values and confidence intervals, we filtered influential VIP > 2.0, as candidate lipid markers. P values less than 0.05 were considered significant and values less than 0.01 were considered highly significant. Variance analysis and T test were used for the age and lipid index and chi-square test were used for the percentage of gender among the three groups.

## 3. Results and Discussion

### 3.1. Clinical Characteristics

The study population is 55 with 29 hyperlipidemia patients in which 10 had syndrome of stagnation of liver qi and spleen deficiency and 19 did not and 26 were healthy volunteers. Sex among the three groups showed no significant differences (P > 0.05). Age comparison showed differences between normal group and hyperlipidemia group (P = 0.01) whereas no significant differences between the two different syndrome groups of hyperlipidemia group (P = 0.68) indicated that the body was prone to abnormal lipid metabolism with age increasing. The clinical characteristics were shown in Table 2.

### 3.2. Chromatograms in Both Positive and Negative Ion Mode

**Figure 1:**
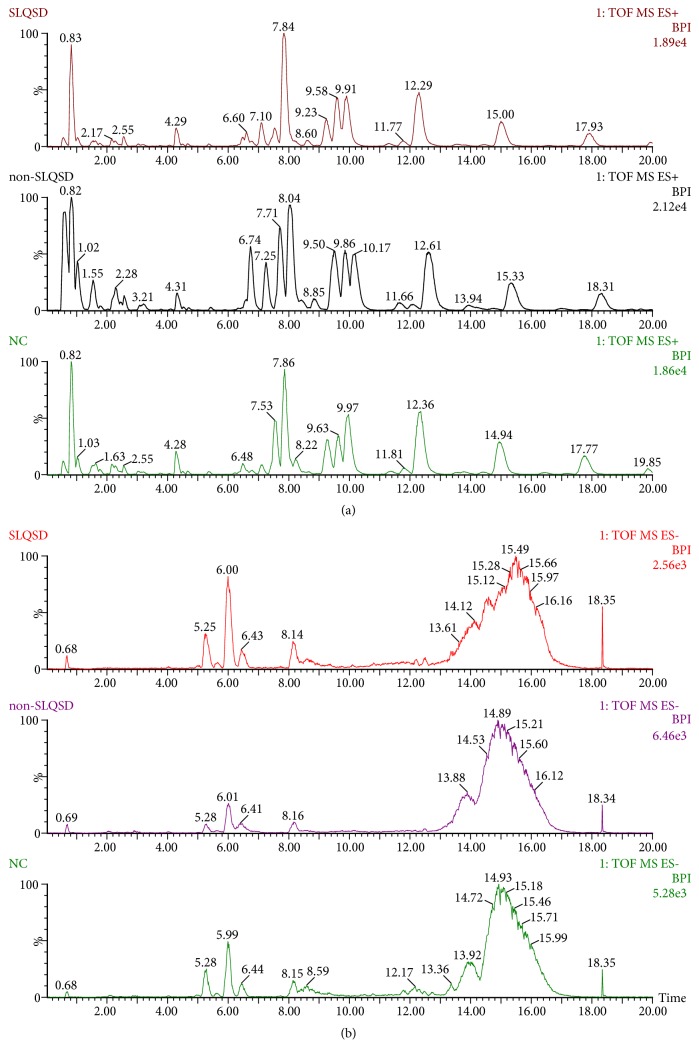
Typical base peak intensity (BPI) chromatograms obtained from plasma of healthy volunteers group (NC), hyperlipidemia with syndrome of non-SLQSD group, and hyperlipidemia with syndrome of SLQSD group in positive ion mode (a) and negative ion mode (b).

As can be seen from Figure 1, there are significant differences in lipid metabolism in both positive and negative ion modes of healthy volunteers and patients with hyperlipidemia. The amounts of mass data obtained in the positive ion mode were more than that in the negative ion mode, indicating that the positive ion mode is more suitable for detecting plasma lipid metabolites. In order to get more comprehensive information on lipid metabolism, we selected both positive and negative ion modes to detect sample.

### 3.3. Plasma Samples Metabolic Profiles

**Figure 2:**
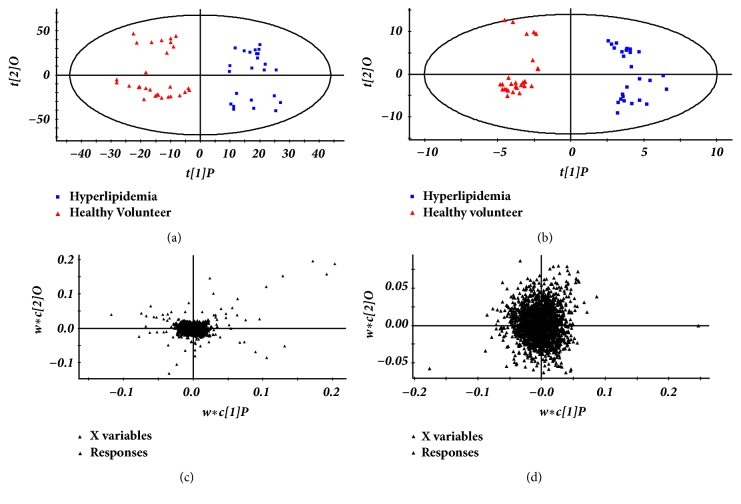
(a) The scores of PLS-DA for healthy volunteers group and hyperlipidemia group in positive ion mode [R2Y(cum)=0.992, Q2(cum)=0.604]. (b) The scores of PLS-DA for healthy volunteers group and hyperlipidemia group in negative ion mode [R2Y(cum)=0.988, Q2(cum)=0.503]. (c) The loading plots derived from UPLC-QTOF/MS data for plasma samples of hyperlipidemia group and healthy volunteers group in positive ion mode. (d) The loading plots derived from UPLC-QTOF/MS data for plasma samples of hyperlipidemia group and healthy volunteers group in negative ion mode.

The subtle changes could be found using a pattern recognition approach, such as PCA and PLS-DA. The supervised PLS-DA model was used to separate plasma sample into two blocks between patients with hyperlipidemia and healthy volunteers (Figure 2).

**Figure 3:**
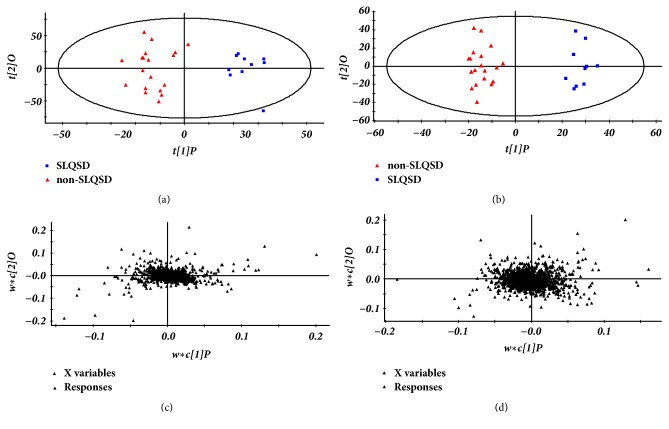
(a) The scores of PLS-DA for hyperlipidemia with syndrome of SLQSD group and hyperlipidemia with syndrome of non-SLQSD group in positive ion mode [R2Y(cum)=0.996, Q2(cum)=0.722]. (b) The scores of PLS-DA for hyperlipidemia with syndrome of SLQSD group and hyperlipidemia with syndrome of non-SLQSD group in negative ion mode [R2Y(cum)=0.85, Q2(cum)=0.719]. (c) The loading plots derived from UPLC-QTOF/MS data for plasma samples of hyperlipidemia with syndrome of SLQSD group and hyperlipidemia with syndrome of non-SLQSD group in positive ion mode. (d) The loading plots derived from UPLC-QTOF/MS data for plasma samples of hyperlipidemia with syndrome of SLQSD group and hyperlipidemia with syndrome of non-SLQSD group in negative ion mode.

The supervised PLS-DA divided samples into two blocks and this method was applied to obtain a better discrimination between the two groups. Based on the differences in their metabolic profiles, the PLS-DA score plot analysis distinguished the plasma samples of hyperlipidemia patients with syndrome of SLQSD and hyperlipidemia patients with syndrome of non-SLQSD (Figure 3).

### 3.4. The Differential Lipids between Different Groups

**Figure 4:**
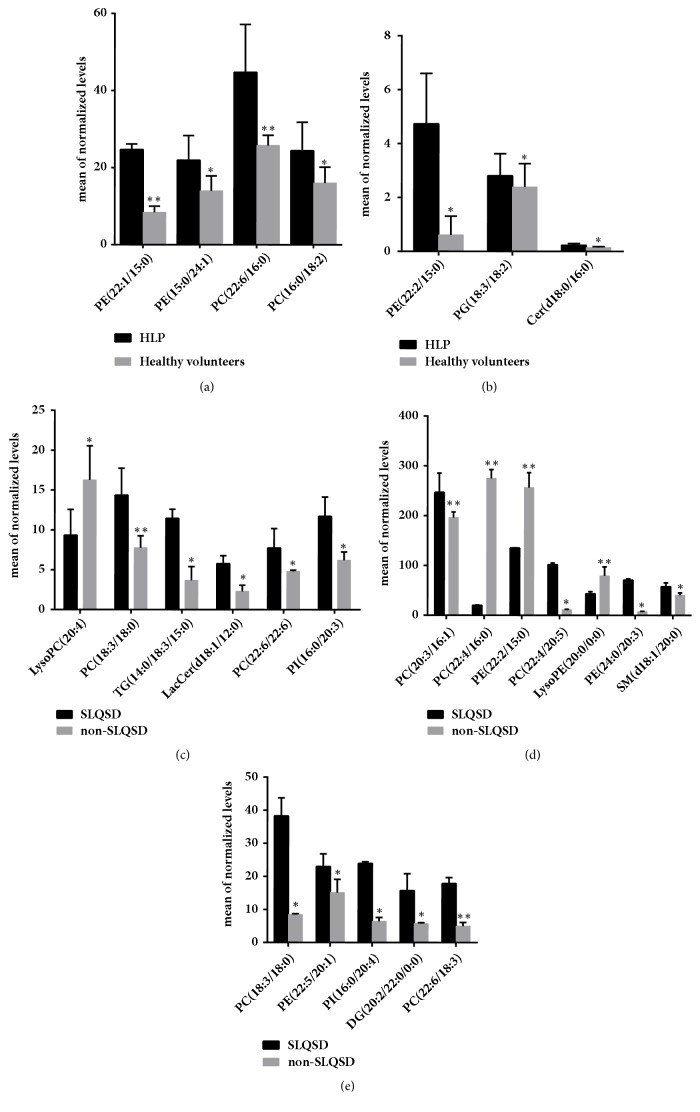
The statistical results of 25 biomarkers. (a, b) Comparison of 7 biomarkers peak relative signal intensities in hyperlipidemia and healthy volunteer. (c-e) Comparison of 18 biomarkers peak relative signal intensities in hyperlipidemia with syndrome of SLQSD and hyperlipidemia with the syndrome of non-SLQSD groups. Values are means ±SD, *∗*P<0.05, and *∗∗*P<0.01.

28 endogenous plasma lipid metabolites, contributing to the separation between the groups, were identified based on their molecular ion information as well as the fragments of corresponding product ion. The identification of the Biomarker was submitted for database searching, either in-house or using the online Scripps Center for Metabolomics database (https://metlin.scripps.edu/), Lipid Maps (http://www.lipidmaps.org/), HMDB (http://www.hmdb.ca), and Chemspider (http://www.chemspider.com) data source. The variables (ions) were identified based on the metabolite identification strategy, and VIP values was also used for the selection of biomarkers (listed in Tables 3 and 4, Fact of Change>2 or <1). Compared with the healthy volunteers, the hyperlipidemia patients had higher concentrations of PC(16:0/18:2), PG(18:3/18:2), Cer(d18:0/16:0), PE(22:1/15:0), PE(15:0/24:1), PC(22:6/16:0) (Table 3 and Figure 4).

Our results indicate that there were a great many differences of lipid metabolism between different syndrome of the same disease, hyperlipidemia, and showed more obvious differences of the main syndrome of SLQSD. Comparing the hyperlipidemia with syndrome of non-SLQSD patients, the hyperlipidemia with syndrome of SLQSD patients exhibited elevated lipid metabolites including PE(22:2/15:0), PC(18:3/18:0), TG(14:0/18:3/15:0), LacCer(d18:1/12:0), PC(20:3/16:1), PC(18:3/18:0), SM(d18:1/20:0), PE(15:0/22:2), PC(22:6/22:6), DG(20:2/22:0/0:0), PE(22:5/20:1), PC(22:6/18:3), PE(24:0/20:3), PI(16:0/20:3), PC(22:4/20:5), and PI(16:0/20:4) (Table 4 and Figure 4).

**Figure 5:**
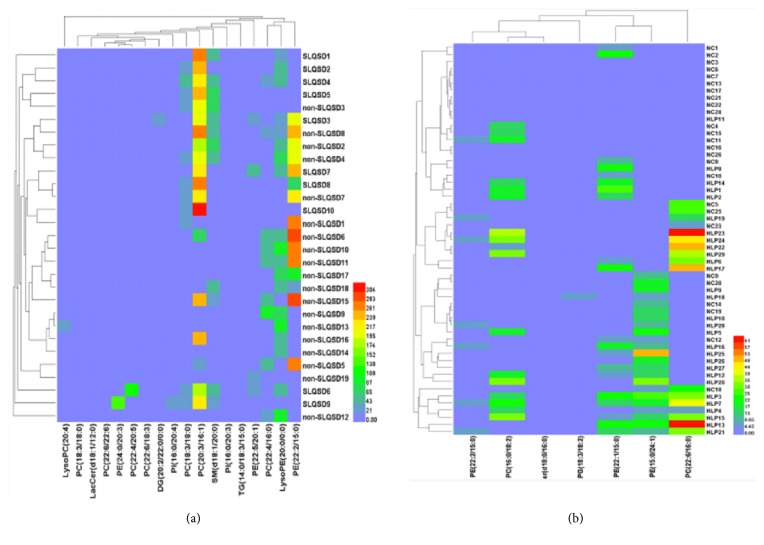
Hierarchical clustering and diagnostic potential of hyperlipidemia metabolite composition. (a) Hierarchical clustering of the plasma metabolome from hyperlipidemia with syndrome of SLQSD and hyperlipidemia with the syndrome of non-SLQSD samples. (b) Hierarchical clustering of the plasma metabolome from hyperlipidemia and healthy volunteer (NC) samples.

According to the differences of metabolites, the different metabolic differences between sample content changes in each group were visualized. As shown in Figure 5, red indicated the higher level of the metabolites. The blue indicated the lower level of the metabolites.

## 4. Discussion

Under Chinese medicine principle guidance, TCM has been widely used in the clinic and has been considered an alternative therapy for the treatment of various diseases, such as hyperlipidemia, diabetes, hypertension, cardiovascular disease, kidney disease, and gastrointestinal disease [26–32]. TCM syndrome is the comprehensive analysis of clinical information gained by the four main diagnostic TCM procedures, observation, listening, questioning, and pulse analysis [33], and is built on the bases of long-term and substantial clinical practice [34]. The complete TCM process is known as Bian Zheng Lun Zhi [35]. TCM treatment is based on the traditional diagnose method to distinguish the TCM syndrome, not the disease. In the development process, TCM diagnosis and treatment system form two systems: disease differentiation and syndrome differentiation [36]. So there is a phenomenon in the relationship between TCM syndrome and disease, called different TCM syndrome for same disease [37]. Researchers used various means to research and explore the essence or modern scientific connotation of TCM syndrome [34, 38].

SLQSD contains nerve, digestion, absorption, metabolism, immune, endocrine, nucleotide, matrix metalloproteinase, blood fluid rheology, and other aspects of change. Due to the complexity and integrity of the syndrome, it is difficult to use a single physiological and biochemical indicator to reveal its essence. So we used lipidomics technology to investigate the syndrome of the modern diseases (hyperlipidemia). The data demonstrated that PLS-DA showed a significantly separation between hyperlipidemia patients and healthy volunteers with the different lipids including PC, PE, PG, and Cer as well as between the hyperlipidemia patients with syndrome of SLQSD and the syndrome of non-SLQSD with the different lipids including PC, PE, PI, TG, DG, SM, LysoPC, LysoPE, and LacCer as shown in Tables 1 and 2. Interestingly, we found that PE(24:0/20:3) has a value of VIP more than 36, while and PC(22:4/20:5) elevated more than 23 folds between the SLQSD and non-SLQSD. The current study demonstrated many differences in lipid metabolism between different syndromes of the same disease such as hyperlipidemia and showed more obvious differences of the main syndrome of SLQSD.

Different types of lipids play different roles in the human body as phosphatidylethanolamine (PE) and phosphatidylcholine (PC) play crucial roles in the biological system to maintain the cellular environmental condition [39]. Oxidative stress and inflammation play a central part in the pathogenesis and progression of various diseases. Oxidative stress targets these phospholipids containing polyunsaturated fatty acids and accompanies the oxidized phospholipids [40–43]. Recent studies have suggested that oxidized phospholipids is associated with inflammation and might induce the atherosclerosis formation by the uptake of oxidized LDL through scavenger receptor as ligands [44]. Accumulated evidence has demonstrated that PC could improve insulin sensitivity and contribute to both proliferative growth and programmed cell death [45]. PC is also the biosynthetic precursor of lysoPC [13]. A number of studies have shown that lysoPC plays a critical role in glucose metabolism, lysoPC activates adipocyte glucose uptake and lowers blood glucose levels in murine models of diabetes [46], and the decreased plasma level of lysoPCs was found in Type 2 diabetes [47]. LysoPE, known as a relational protein, is involved in several motility-related processes such as angiogenesis and neurite outgrowth [48]. Glycosphingolipids are known to interfere with insulin signaling at elevated levels [49]. Lactosylceramide is highly expressed on the plasma membranes of human phagocytes and mediates several immunological and inflammatory reactions, including phagocytosis, chemotaxis, and superoxide generation [50]. Other studies proved that SLQSD mainly involves the decrease of thymus function and insufficient release of cytokines at early immune response stage and also involves the inhibition of cellular immunity and humoral immunity [51]. Cer(d18:0/16:0) has high sensitivity and specificity on the prognosis related to major adverse cardiovascular events after ST-segment elevation myocardial infarction [52].

The current study not only indicated that lipidomics was an effective method to distinguish different TCM syndromes of hyperlipidemia but also showed the changing trend of lipid metabolites between different syndromes. Future researches will focus on the discovery of specific lipid such as PE(24:0/20:3) and PC(22:4/20:5) of syndrome of SLQSD in other diseases and the validation of the explorative biomarkers. In addition, more efforts will be directed to the biological interpretation including investigating which pathway is involved in the lipids changes associated with the onset, development, and progression of hyperlipidemia and whether these changes are the same during onset and progression, or whether different changes of lipids occur of different syndrome. In the future, large sample studies are needed to reveal whether the biological basis of SLQSD is the oxidative stress and inflammatory reaction caused by PC(22:4/20:5) and PE(24:0/20:3). In addition to the clinical detection indicators of blood lipid, we need to know whether other lipids such as PC, PE, PG, and Cer can be new or early diagnostic indicators of dyslipidemia. Combined with systems biology and other techniques, it is possible to analyze the biological biomarkers of TCM syndrome more comprehensively.

## Figures and Tables

**Table 1:** Linear gradient composition.

Time (min)	Water (0.1% formic acid, 1mol/L ammonium acetate)	Acetonitrile
0	65	35
3	45	55
15	0	100
17	0	100
17.1	65	35
20	65	35

**Table 2:** Clinical characteristics.

Clinical characteristics	hyperlipidemia with syndrome of SLQSD	hyperlipidemia with syndrome of non-SLQSD	Healthy volunteers
n	10	19	26
Age (X±SD)	55.6±9.64*∗*	54.26±8.05*∗∗*	46.73±7.74
Gender [Female (%)]	60.0	63.2	53.8
TC	4.90±0.69*∗∗*^##^	5.88±0.72*∗∗*	4.37±0.60
TG	2.24±0.84*∗∗*	2.00±1.27*∗∗*	1.02±0.26
HDL-C	1.17±0.31	1.40±0.33	1.37±0.27
LDL-C	2.80±0.56^##^	3.55±0.75*∗∗*	2.50±0.48
VDL-C	0.96±0.25*∗∗*	0.93±0.50*∗∗*	0.49±0.14
ApoA_1_	1.36±0.30	1.44±0.19	1.40±0.18
ApoB	1.02±0. 25	1.15±0. 31*∗∗*	0.83±0. 16

Gender was expressed as percentage and the other data were expressed as mean ± SD. *∗*p<0.05 and *∗∗*p< 0.01 compared with healthy volunteer group. Hyperlipidemia with syndrome of SLQSD compared with hyperlipidemia with syndrome of non-SLQSD, ^#^p<0.05, and ^##^p < 0.01.

**Table 3:** Identification results of varying ions and their change trend of hyperlipidemia and healthy volunteers.

NO	Retention Time	Mass	Group 1 Content	Group 2 Content	Factor of Change	VIPvalue	Change Trend	Identified potential Biomarker	Lipid Class	Ion
1	8.03	758.5651	4.7339±1.869*∗*	0.61515±0.6942	7.7	4.76901	↑	PE(22:2/15:0)	Glycerophospholipid	M+H
2	14.1	758.5656	24.4239±7.3675*∗*	16.0367±4.1211	1.5	19.6218	↑	PC(16:0/18:2)	Glycerophospholipid	M+H
3	15.4	769.5915	2.8041±0.8225*∗*	2.3896±0.8705	1.2	7.40167	↑	PG(18:3/18:2)	Glycerophospholipid	M+H
4	16.15	540.4826	0.2268±0.0674*∗*	0.1447±0.0368	1.6	4.95507	↑	Cer(d18:0/16:0)	Sphingomyelin	M+H
5	18.05	760.5777	24.6892±1.4525*∗∗*	8.4016±1.6207	2.9	16.1507	↑	PE(22:1/15:0)	Glycerophospholipid	M+H
6	14.32	832.6021	21.9211±6.4255*∗*	13.9741±3.9031	1.6	4.2825	↑	PE(15:0/24:1)	Glycerophospholipid	M+FA-H
7	14.37	804.5628	44.7529±12.4215*∗∗*	25.7829±2.6471	1.7	2.48245	↑	PC(22:6/16:0)	Glycerophospholipid	M+FA-H

Group 1: hyperlipidemia. Group 2: healthy volunteers.↑ indicated the concentrations compared to the other group are increasing. ↓ indicated the concentrations compared to the other group are reducing. *∗*P<0.05 and *∗∗*P<0.01. PE: phosphatidylethanolamine; PC: phosphatidylcholine; PG: phosphatidylglycerol; Cer: ceramide.

**Table 4:** Identification results of varying ions and their change trend of hyperlipidemia with syndrome of SLQSD and hyperlipidemia with the syndrome of non-SLQSD.

NO	Retention Time	Mass	Group 3Content	Group 4 Content	Factor of change	VIP value	Change Trend	Identified potential Biomarker	Lipid Class	Ion
1	5.53	544.3342	9.3314±3.2342*∗∗*	16.2656±4.2760	0.6	7.2994	↓	LysoPC(20:4)	Lysophospholipid	M+H
2	12.28	784.5734	14.3592±3.3714*∗*	7.7689±1.4901	1.8	10.0211	↑	PC(18:3/18:0)	Glycerophospholipid	M+H
3	12.34	787.5918	11.4418±1.1513*∗*	3.6700±1.7245	3.1	6.0391	↑	TG(14:0/18:3/15:0)	Glycerolipids	M+H
4	12.64	806.5652	5.7728±1.0044*∗*	2.2932±0.7673	2.5	6.1186	↑	LacCer(d18:1/12:0)	Sphingomyelin	M+H
5	15.81	782.5518	246.9174±38.6396*∗∗*	196.1073±11.3457	1.3	63.2972	↑	PC(20:3/16:1)	Glycerophospholipid	M+H
6	16.48	784.5824	38.2776±5.4524*∗∗*	8.4079±0.3322	4.6	16.5178	↑	PC(18:3/18:0)	Glycerophospholipid	M+H
7	16.69	759.5692	57.5260±7.8236*∗*	40.4171±4.7738	1.4	12.2461	↑	SM(d18:1/20:0)	Sphingolipid	M+H
8	16.86	810.5915	19.9225±0.8748*∗∗*	274.9301±17.5735	0.1	2.44919	↓	PC(22:4/16:0)	Glycerophospholipid	M+H
9	18.30	758.5560	135.1895±8.1870*∗∗*	256.3050±30.1737	0.5	36.2027	↓	PE(22:2/15:0)	Glycerophospholipid	M+H
10	8.50	508.3418	43.0992±4.4230*∗∗*	79.1179±18.2387	0.5	3.03607	↓	LysoPE(20:0/0:0)	Lysophospholipid	M-H
11	14.32	876.5754	7.7353±2.4394*∗*	4.7881±0.1727	1.6	2.02951	↑	PC(22:6/22:6)	Glycerophospholipid	M-H
12	14.38	351.2478	15.6962±5.1028*∗*	5.7276±0.27980	2.8	2.29853	↑	DG(20:2/22:0/0:0)	Glycerolipids	M-2H
13	15.05	800.5540	22.9620±3.8468*∗*	15.0764±4.0387	1.5	2.17423	↑	PE(22:5/20:1)	Glycerophospholipid	M-H_2_0-H
14	15.3	826.6140	17.8481±1.8121*∗∗*	4.9814±1.1049	3.6	1.14469	↑	PC(22:6/18:3)	Glycerophospholipid	M-H
15	15.94	852.6908	17.8481±1.8121*∗∗*	4.9814±1.1049	3.6	2.44998	↑	PE(24:0/20:3)	Glycerophospholipid	M-H
16	16.00	841.5830	11.7030±2.4241*∗*	6.2152±1.0354	1.9	1.77427	↑	PI(16:0/20:3)	Phosphatidylinositol	M-H_2_0-H
17	16.25	854.6047	101.6059±23.4842*∗*	11.3739±0.8854	8.9	2.41923	↑	PC(22:4/20:5)	Glycerophospholipid	M-H
18	16.45	857.5742	23.9200±0.5182*∗*	6.4069±1.1494	3.7	1.47236	↑	PI(16:0/20:4)	Phosphatidylinositol	M-H

Group 3: hyperlipidemia with syndrome of SLQSD and Group 4: hyperlipidemia with the syndrome of non-SLQSD. ↑ indicated the concentrations compared to the other group are increasing, and ↓indicated the concentrations compared to the other group are reducing. *∗*P<0.05 and *∗∗*P<0.01. PE: phosphatidylethanolamine; PC: phosphatidylcholine; TG: triglyceride; DG: diglyceride; PI: phosphatidylinositol; SM: sphingomyelin; LysoPC: lysophosphatidylcholine; LysoPE: lysophosphatidylethanolamine.

## Data Availability

The data used to support the findings of this study are available from the corresponding author upon request.
